# Mechanisms Underlying the Effects of Secretory Protein *G22* on Biological Characteristics and Virulence of *Streptococcus suis*

**DOI:** 10.3390/microorganisms13040774

**Published:** 2025-03-28

**Authors:** Shiyue Fan, Yanping Tan, Zhiwei Li, Yanyan Zhang, Jinquan Li, Ye Feng, Yi He, Xiaoling Chen, Xingxing Dong

**Affiliations:** 1 National R&D Center for Serich Agricultural Products Processing, School of Modern Industry for Selenium Science and Engineering, Wuhan Polytechnic University, Wuhan 430023, China; fanshiyue_0612@163.com (S.F.); 15395560807@163.com (Y.T.); heyi629@126.com (Y.H.); 2 Joint International Research Laboratory of Animal Health and Animal Food Safety, College of Veterinary Medicine, Southwest University, Chongqing 400715, China; zhiweili@stanford.edu; 3 Division of Infectious Diseases and Geographic Medicine, Department of Medicine, Stanford University, Stanford, CA 94305, USA; 4 Engineering Research Center of Feed Protein Resources on Agricultural By-Products, Ministry of Education, Wuhan Polytechnic University, Wuhan 430023, China; zhangyanyana305@163.com; 5 State Key Laboratory of Agricultural Microbiology, College of Biomedicine and Health, Huazhong Agricultural University, Wuhan 430070, China; lijinquan2007@gmail.com; 6 State Key Laboratory of Agricultural Microbiology, College of Food Science and Technology, Huazhong Agricultural University, Wuhan 430070, China; 7 Institute of Translational Medicine, Zhejiang University School of Medicine, Hangzhou 310029, China; pandafengye@zju.edu.cn

**Keywords:** *Streptococcus suis*, *G22*, bacterial virulence, gene knockout, transcriptome sequencing

## Abstract

*Streptococcus suis* serotype 2 (SS2) is an important zoonotic pathogen that seriously harms the swine industry and human health. However, its pathogenic mechanisms are largely unknown, and the few virulence factors reported so far are insufficient to systematically explain its infectious and pathogenic mechanisms. In preliminary research, we identified a gene named *G22* encoding a hypothetical secreted protein that may be closely associated with the high-level pathogenicity of *S. suis*. In this study, we constructed deletion and complementation strains of the *G22* gene through homologous recombination and explored its roles in the pathogenicity and susceptibility of *S. suis* to environmental stresses through in vitro and in vivo experiments. The deletion of *G22* clearly influenced the typical capsular structure of SS2 and impaired the bacterium’s growth in a medium containing hydrogen peroxide (showing a growth reduction of 32.98% ± 5.23% compared to the wild-type strain SC19, *p* < 0.001) or with a low pH (with a growth inhibition of 17.44% ± 1.9% relative to the wild-type strain SC19, *p* < 0.01). Δ*G22* also showed reduced survival in whole blood and in RAW 264.7 macrophages (with a survival reduction of 16.44% ± 2.29% compared to the wild-type, *p* < 0.001). The deletion of *G22* also sharply attenuated the virulence of SS2 in a mouse infection model (reducing the mortality rate by 50% ± 0.04%, *p* < 0.05). We also demonstrated that *G22* is required for the adhesion and invasion of SS2 in host cells. An RNA sequencing analysis revealed that 50 genes were differentially expressed in the Δ*G22* and wild-type strains: 23 upregulated and 37 downregulated. Many of the genes are involved in carbohydrate metabolism and the synthesis of virulence-associated factors. Several genes associated with the phosphotransferase system were significantly upregulated in strain Δ*G22*. In summary, *G22* plays a role in the morphological development and pathogenesis of the highly virulent SS2 strain SC19.

## 1. Introduction

*Streptococcus suis* (*S. suis*) is an important emerging zoonotic agent that causes septicemia, meningitis, endocarditis, arthritis, septicemia, meningitis, and even sudden death in humans. Although *S. suis* is considered a major swine pathogen, it is increasingly being isolated from a wide range of mammalian species and from birds [1]. *S. suis* has spread worldwide and has caused enormous economic losses in the swine industry in recent years because it causes high morbidity and mortality [2]. *S. suis* infections in humans are most often restricted to workers in close contact with pigs or swine byproducts [3]. However, two extensive outbreaks of SS2 in humans in China in 1998 and 2005 raised serious concerns for public health and changed our perception that human SS2 infections were only sporadic [1,4]. Almost all of the known strains of SS2 mainly infect swine and humans [5]. Serotype 2 is the serotype most frequently isolated from diseased pigs in the majority of countries [6], and most research has focused on this serotype [5]. However, the molecular pathogenesis of *S. suis*-induced infectious disease is still unclear, and our understanding of it is fragmentary [4,7], which hampers our attempts to control the diseases caused by *S. suis* [8].

A repertoire of *S. suis* virulence determinants undoubtedly plays a role in human infections because *S. suis* has been suggested to cause community-acquired disease [9]. Many SS2 virulence factors have been reported, some of which are considered critical, including glyceraldehyde 3-phosphate dehydrogenase (Gapdh), suilysin (Sly), Eno, and capsular polysaccharide (Cps) [10]. For example, a deficiency in O-acetyl-homoserine sulfhydrylase (OAHS) reduces the expression of Eno and thus inhibits SC19-induced endothelial cell apoptosis, ultimately alleviating the damage caused by SC19 to the blood–brain barrier (BBB). Although the virulence of SC19-ΔOAHS is significantly reduced, it still induces a good immune response in mice against infection by a homologous strain [11]. Cps not only forms the basis for serotyping but also protects the bacterium from phagocytosis [12]. Catabolite control protein A (CcpA) is involved in metabolic gene regulation in various bacteria [13] and is the major mediator of carbon catabolite repression (CCR), repressing gene expression in response to excess sugar during growth [14,15,16]. Bacterial growth, hemolysin production, biofilm formation, and capsule expression are also influenced by CcpA depletion in other streptococci [17,18]. CcpA only indirectly affects certain pathogenic proteins, thus reducing virulence [19]. Although several virulence factors have been shown to play roles in the early stages of infection, the roles of other virulence factors remain unclear [20]. Therefore, further investigations into the pathogenesis of known and unknown virulence factors will extend our understanding of the pathogenesis of, and prevent infection by, this bacterium [21].

In a previous study, we analyzed the genomic sequences of 1634 *S. suis* isolates from 14 countries and classified them into nine Bayesian analysis of population structure (BAPS) groups. Among them, BAPS group 7 represented a dominant group of virulent *S. suis* associated with human infections, which were most commonly sequence type 1 (ST1) and ST7 (included in clonal complex 1 [CC1]). We proposed that this cluster constituted a novel human-associated clade (HAC) that had diversified from swine *S. suis* isolates. A genome-wide association study was used to identify 25 HAC-specific genes. These genes may contribute to an increased risk of human infection and could be used as markers for HAC identification [22]. The *G22* gene, annotated as a secretory protein gene, was one of these 25 HAC-specific genes. Based on these findings, we hypothesize that the *G22* protein plays a crucial role in the pathogenicity of SS2 and that its inactivation may lead to the attenuation of virulence. Specifically, we propose that the *G22* protein contributes to the virulence of *S. suis* by modulating the expression of key virulence factors and influencing host–pathogen interactions. To test this hypothesis and clarify the biological functions of the *G22* protein, we used homologous recombination to construct a gene knockout mutant, Δ*G22*, of SC19. Comprehensive experiments showed clear morphological changes and the attenuation of pathogenicity in the mutant strain. An RNA sequencing analysis suggested that the inactivation of *G22* resulted in the up- or downregulation of many genes involved in carbohydrate metabolism or encoding virulence-related factors. Our results provide new insights into the pathogenesis of SS2 and extend our understanding of this pathogen.

## 2. Materials and Methods

### 2.1. Bacterial Strains and Plasmids

The *S. suis* strain and plasmids used in this study are given in Table 1. In this study, we used SS2 strain SC19, one of the representative Chinese virulent strains that were isolated from a diseased pig during an outbreak in the Sichuan province of China [23]. The SS2 strains were cultured at 37 °C in tryptic soy broth (TSB) or on tryptic soy agar (Difco, Detroit, MI, USA) (TSA) plates containing 10% newborn bovine serum (Sijiqing Biological Engineering Materials Co., Ltd., Hangzhou, China). *E. coli* strains were cultured on Luria–Bertani (LB) broth or LB agar plates at 37 °C. During selection procedures, spectinomycin (Spc; Sigma-Aldrich, St. Louis, MO, USA) was added at concentrations of 100 μg/mL for *S. suis* and 50 μg/mL for Escherichia coli to maintain plasmid stability. DMEM culture medium (Gibco, Invitrogen, Carlsbad, CA, USA) including 10% fetal calf serum (Gibco, Carlsbad, CA, USA) was used to culture the human laryngeal epithelial cell line (HEp-2) and Raw264.7 macrophage cell line at 37 °C in a 5% CO_2_ humidified atmosphere. SS2 strain SC19 was selected as the wild-type (WT) strain. Strain SC19 and the *G22*-deletion mutant ∆*G22* were cultured in TSB or on TSA plates containing 10% newborn bovine serum (Sijiqing Biological Engineering Materials Co., Ltd., Hangzhou, China) at 37 °C under 5% CO_2_. When the pSET4s plasmid contained Spc, the concentration of Spc in the medium was 100 µg/mL.

### 2.2. Construction of G22 Gene Knockout Strain and Complementary Strain

To cause the deletion of *G22* in the SC19 strains, we utilized the thermosensitive suicide vector pSET4s, derived from *S. suis-E. coli* [24]. Initially, the specific primers outlined in Table 2 were employed to amplify the two flanking regions adjacent to the target gene. Subsequently, these regions were fused through overlap-extension PCR. Following purification, the resultant PCR products were treated with BamH1 and EcoRI restriction enzymes. Concurrently, the temperature-sensitive vector pSET4s was ligated with the digested PCR fragments to facilitate the cloning of the desired gene. Electroporation was then utilized to introduce the recombinant vectors into competent SS2 SC19 cells. The cultivation of the transformed cells took place at 28 °C for 36 h in media supplemented with Cm and Spc. We subsequently screened for vector-loss mutants, which had undergone homologous recombination via a double-crossover event, replacing their wild-type (WT) allele with a genetic segment harboring the *G22* deletion. The verification of the mutant cells was carried out using PCR with the primer pairs W1/W2 (to differentiate between the WT and mutant based on amplicon size) and N1/N2 (to confirm the absence of *G22*).

For complementation purposes, the target gene, along with its putative promoter sequences, was amplified from the SC19 genome via PCR and cloned into the *E. coli-S. suis* shuttle vector pSET2 [25], generating recombinant plasmids. These plasmids were then electroporated into the Δ*G22* mutant cells. Furthermore, complementation strains (CΔ*G22*) were selected using Spc and validated by PCR. The sequences of all primers utilized in this study are detailed in Table 2.

### 2.3. Growth Characteristics and Genetic Stability of Mutant Strains

The experiment involved the separate inoculation of WT SC19, its mutant derivative Δ*G22*, and the complemented strain CΔ*G22*; all strains reached equivalent viable counts (~10^8^ CFU/mL) at OD*_600_* = 0.6 before the inoculation, each diluted to a 1:100 ratio, into 100 mL aliquots of TSB. These cultures were then incubated at 37 °C with shaking at 180 rpm. To monitor bacterial growth, optical density measurements at a wavelength of 600 nm (OD_600_) were taken using an enzyme-linked immunosorbent assay reader (Smart Spec Plus, Bio-Rad, Hercules, CA, USA) every hour for a duration of 20 h. This experimental setup was repeated across three independent biological replicates to ensure the reliability of the results.

### 2.4. Observation with Transmission Electron Microscopy (TEM)

Transmission electron microscopy (TEM) analyses were conducted following established protocols [26]. Cells from the SS2 strains SC19, Δ*G22*, and CΔ*G22* were collected at an optical density (OD_600_) of 0.6 and subsequently fixed overnight in 2.5% glutaraldehyde. Following this, the samples underwent post-fixation with 2% osmium tetroxide for two hours and were dehydrated through a graded ethanol series. The dehydrated specimens were then embedded in epoxy resin for sectioning. Ultrastructural examinations were performed using an H-7650 TEM instrument (Hitachi, Tokyo, Japan) to assess the morphological features of the bacterial cells.

### 2.5. Survival Assays of SS2 in H_2_O_2_, at High Temperature, and Under Acidic Conditions

To assess stress tolerance, we executed the following methodology. Initially, bacterial suspensions of the WT, Δ*G22* mutant, and complemented CΔ*G22* strains were propagated and adjusted to an OD_600_ nm of 0.6 in TSB medium; all strains reached equivalent viable counts (~10^8^ CFU/mL). Subsequently, 100 μL aliquots of each strain were individually subjected to various stress conditions: heat stress at temperatures of 40 °C, 41 °C, and 42 °C; acid stress mediated by acetic acid at pH 4, pH 5, and pH 6, all maintained at 37 °C; and oxidative stress induced by 10 mM H_2_O_2_, also at 37 °C. These stressed cultures were incubated for 1 h under their respective conditions. Concurrently, untreated strains were incubated at 37 °C for 1 h, serving as negative controls. To quantify the stress-induced survival, serial dilutions of the bacterial suspensions under all conditions were plated onto TSA plates. The colony-forming unit (CFU) counts under each stress scenario were then determined. The survival rate under a given stress was calculated as a percentage, using the formula [(CFU under stress)/(CFU in negative control)] × 100%, adopting a previously established methodology [27]. This experimental protocol was independently replicated three times to ensure reproducibility and statistical robustness.

### 2.6. Adhesion and Invasion Assays in Caco-2 and HEp-2 Cells

We utilized human laryngeal cancer epithelial cells (HEp-2) and human colorectal adenocarcinoma cells (Caco-2) to conduct adhesion and invasion assays for SS2, as described previously, with some modifications [21]. These two cell lines had previously been used to study interactions with SS2 strains [9,28]. These cell lines were purchased from Pricella Biotechnology Co., Ltd. and Warner Biotechnology Co., Ltd. (Wuhan, China), respectively. Strains were streaked on TSA containing 10% fetal bovine serum and incubated for 16 h on 37 °C. The cultures were then used to inoculate fresh TSB containing 10% newborn bovine serum and cultured overnight. For the adhesion assay, bacterial strains were collected at the mid-exponential growth phase (OD_600_ = 0.6) via centrifugation and subsequently washed twice with PBS; all strains reached equivalent viable counts (~10^8^ CFU/mL) before inoculation. The bacteria were then resuspended in a DMEM culture medium lacking antibiotics to achieve a concentration of 5 × 10^7^ CFU/mL before being added to 24-well tissue culture plates containing HEp-2 and Caco-2 cells. The plates were incubated at 37 °C for 2 h. Infected cells were rinsed three times with PBS to remove unbound bacteria. The number of adherent bacteria was determined by plating serial 10-fold dilutions on TSB agar plates and incubating them at 37 °C for 12 h to count the colonies that had formed. The invasion assay procedure was similar to that of the adhesion assay, with the exception that extracellular and surface bacteria were eliminated using streptomycin (100 ng/mL) and penicillin (100 ng/mL). Each assay was carried out in triplicate to ensure reproducibility.

### 2.7. Anti-Phagocytosis Analysis in RAW 264.7 Macrophages

To assess the intracellular survival capacity of bacteria, murine RAW264.7 macrophages were propagated in DMEM medium fortified with 10% FBS, then distributed into 24-well plates at a density of 4 × 10^5^ cells per well, following an adapted protocol from the prior literature [29]. Prior to infection, bacterial cultures in the logarithmic phase were harvested by centrifugation, rinsed twice with sterile PBS, and redispersed in fresh DMEM devoid of serum. The macrophages were subsequently challenged with these bacteria at a multiplicity of infection (MOI) of 100. Following a 2 h co-incubation period at 37 °C under a 5% CO_2_ atmosphere, the macrophages were subjected to extensive washing with PBS and maintained in DMEM supplemented with 1% FBS and antibiotics (streptomycin, 100 ng/mL; penicillin, 100 ng/mL) throughout the experimental duration. To quantify intracellular bacterial survival, infected cells were sampled 2 h post-antibiotic treatment. After triplicate washes with sterile PBS, the macrophages were lysed using 0.02% Triton X-100 (Shanghai Macklin Biochemical Co., Ltd., Shanghai, China) at 37 °C for 15 min, thereby releasing the intracellular bacteria. The resultant lysates underwent serial dilutions and were plated onto TSA agar, followed by overnight incubation at 37 °C. Colony forming units (CFUs) were enumerated to ascertain the intracellular bacterial load. To track the dynamics of bacterial survival over time relative to the initial intracellular bacterial count, a relative CFU (rCFU) metric was employed, calculated as the ratio of CFUs at a given time point (x) to the CFUs at the starting time point (0), adapted from the methodology described by Cumley [30]. This experimental design incorporated three independent biological replicates, each replicated technically in triplicate to ensure robust statistical analysis.

### 2.8. Whole-Blood Bactericidal Assay

The survival of SS2 in whole blood was determined as described in previous studies [31]. Briefly, each bacterial suspension was incubated at 37 °C for 5 h under static conditions and then centrifuged at 6000 rpm for 5 min. The pellets were suspended in PBS and the microbial concentrations adjusted to 10^4^ CFU/mL. SC19 (100 µL) or an equal volume of ∆*G22* and C∆*G22* was added to 900 µL of fresh heparinized pig blood from clinically healthy pigs for 3 h at 37 °C. The initial bacterial volume was set to 100%, and the percentage of remaining bacteria was recorded at this time point (after 3 h). Each assay was performed as three independent biological replicates.

### 2.9. Experimental Infection of Mice

Overnight cultures of SS2 strains SC19 and ∆*G22* were washed twice with PBS and suspended in PBS. Twenty-four 6-week-old female BALB/c mice were randomly divided into three groups, with eight mice in each. They were challenged with 200 µL of PBS containing 5 × 10^8^ colony-forming units (CFUs) of SC19 or ∆*G22* or with 200 µL of PBS as the blank control. The infected mice were monitored daily for 7 days. Clinical symptoms and deaths were observed and recorded postinfection. Another 18 BALB/c mice were divided into three groups and injected intraperitoneally with 5 × 10^7^ CFU per mouse, to examine the invasion and colonization capacities of SS2 strains SC19 and ∆*G22*. At 24 h postinfection, the infected mice were killed, and brain, blood, spleen, lung, and liver samples were collected. The numbers of colonizing bacteria were measured by plating diluted samples onto TSA containing 10% newborn bovine serum. The present study was carried out in strict adherence with the ethical standards for animal experimentation set forth by the Institutional Animal Care and Use Committee (IACUC) of Hubei Academy of Preventive Medicine/Hubei Provincial Center for Disease Control and Prevention. The experimental protocol was meticulously reviewed and approved by the IACUC, with the assigned ethics approval number 202310190. Throughout the research, we upheld the highest standards of animal care and welfare, ensuring that all procedures were consistent with national and international guidelines on the humane treatment of animals. This included the implementation of measures to alleviate pain and distress, such as the appropriate use of anesthesia and analgesia, as well as the application of the 3R principle—Replacement, Reduction, and Refinement—to optimize scientific rigor while minimizing animal usage.

### 2.10. RNA Sequencing Analysis

The total RNAs of WT and Δ*G22* cultures were extracted in the late exponential phase with the Total RNA Isolation Kit (Shanghai Sangon Biotech, Shanghai, China). RNA libraries were constructed and Illumina sequencing was performed on an Illumina HiSeq™ sequencer (Novogene, Beijing, China). The complete genomic sequence of SC19 (accession number: CP020863) was used as the reference genome against which the processed reads from each sample were aligned with Bowtie2. The DESeq R package (1.18.0) was used to identify the differentially expressed genes (DEGs). The DEGs were functionally annotated with the NCBI, ENSEMBL, Gene Ontology (GO), UniProt, and Kyoto Encyclopedia of Genes and Genomes (KEGG) databases and a NCBI BLAST+ (version 2.13.0) alignment analysis.

### 2.11. Statistical Analysis

Statistical analyses were conducted using GraphPad Prism 8.3.0 software (GraphPad Software, San Diego, CA, USA). Data are presented as the mean ± standard error of the mean (SEM). Appropriate statistical tests were selected based on the nature of the data and the experimental design. For normally distributed data with equal variances, one-sample *t*-tests or unpaired *t*-tests were used to compare differences between two groups. For comparisons among multiple groups, one-way ANOVA was employed. When data did not meet the assumptions of normality or equal variances, non-parametric tests such as the Mann–Whitney U test or Kruskal–Wallis test were utilized. For in vivo virulence experiments, survival was analyzed using the LogRank test. All statistical tests were two-tailed, and a *p*-value < 0.05 was considered statistically significant.

## 3. Results

### 3.1. Identification of G22 Gene Knockout Strain and Complementary Strain

**Figure 1:**
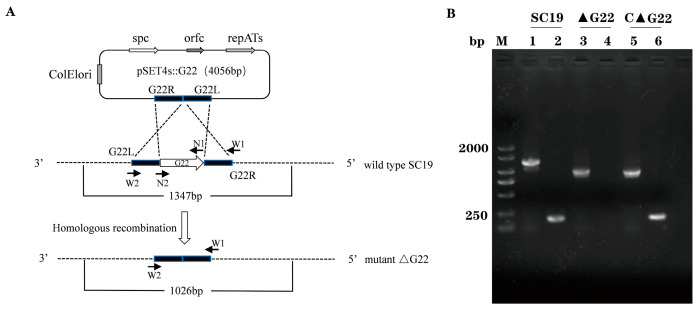
Identification of *G22* gene knockout strain and complementary strain. (**A**) *G22* knockout technology roadmap. *G22*L: Upstream fragment amplification of *G22*. *G22*R: Downstream fragment amplification of *G22*. W1/W2: Identification outside the homologous region. N1/N2: Identification of the internal fragment of *G22*. (**B**) Confirmation of the ∆*G22* mutant (M, DNA Marker, Lanes 1, 3, and 5 were amplified with the external primers W1/W2. Lanes 2, 4, and 6 were amplified with the internal primers N1/N2).

The deletion of the *G22* gene was verified with PCR using the primers listed in Table 1. As shown in Figure 1B, a large fragment (1347 bp) was amplified from the SC19 genome with the external primers (W1/W2) of *G22*, and a small genome fragment (1026 bp) was amplified from the ∆*G22* and C∆*G22* genome. The fragments in ∆*G22* were smaller than those from the parent strain SC19 (Figure 1; Supplementary Materials, Figure S1). A small fragment (202 bp) was amplified from the SC19 and C∆*G22* genome with the internal primers (N1/N2) of *G22*. And no fragment was amplified from the ∆*G22* genome with the internal primers of *G22* (Figure 1B). In summary, we successfully deleted the *G22* gene.

### 3.2. Characterization of Mutant Strain ∆G22

**Figure 2:**
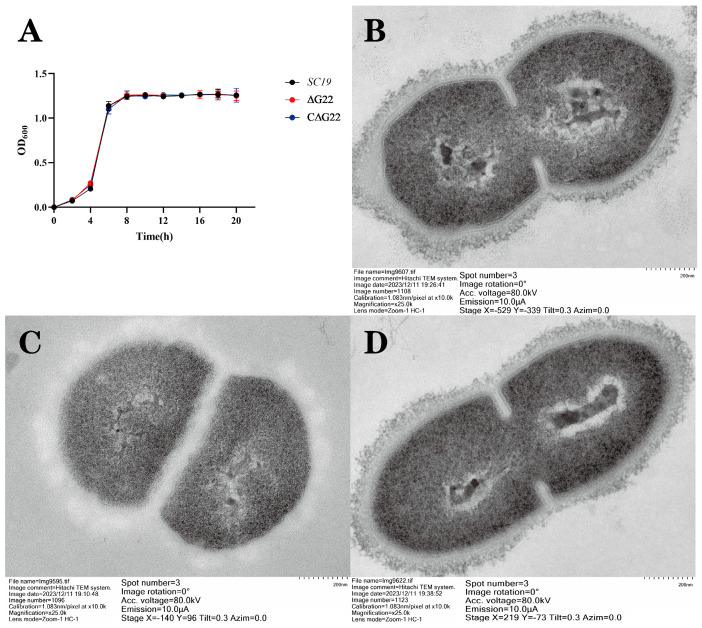
(**A**) Growth characteristics of SC19, ∆*G22*, and C∆*G22*. Transmission electron micrographs of SC19 (**B**), ∆*G22* (**C**), and C∆*G22* (**D**).

We examined the phenotypes of ∆*G22* and C∆*G22*. Growth curves indicated few differences between ∆*G22*, C∆*G22*, and SC19 (Figure 2A), so the deletion of the *G22* gene did not greatly affect the growth characteristics of ∆*G22*. To investigate whether Cps (cellular polysaccharide) was altered by *G22* deficiency, we examined the mutant ∆*G22*, complemented C∆*G22*, and SC19 strains with TEM. The Cps structure and composition of SC19 and C∆*G22* are basically consistent (Figure 2B,D). Surprisingly, the Cps structure and composition of ∆*G22* differ markedly from those of SC19 (Figure 2C). The Cps structure of ∆*G22* shows irregular matrix deposition with visible spatial discontinuities, indicating that the *G22* protein is involved in the synthesis or regulation of Cps.

### 3.3. Role of G22 in Oxidative Stress Tolerance of SS2

**Figure 3:**
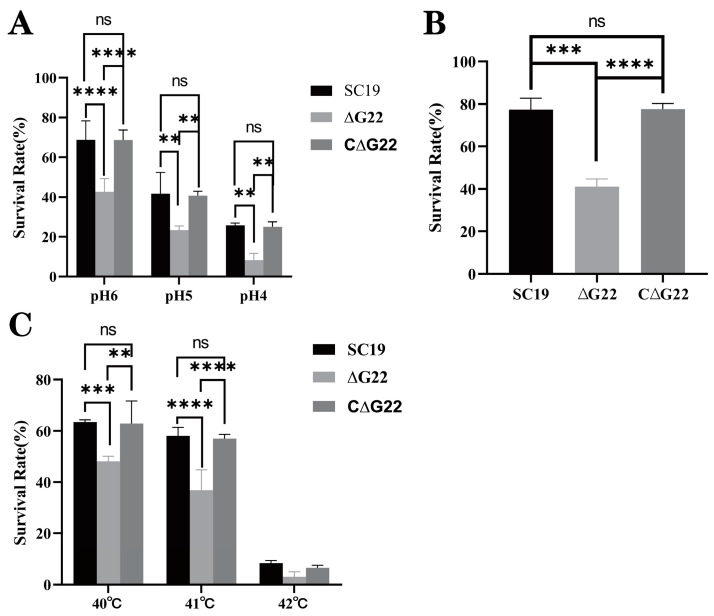
Survival capacities of SC19, ∆*G22*, and C∆*G22* under stress imposed by acid (**A**), H_2_O_2_ (**B**), or high temperatures (**C**). Data represent the mean ± SEM of at least three independent experiments. ‘ns’, ‘**’, ‘***’, and ‘****’ indicate significant difference values with ‘*p* > 0.05, *p* < 0.01, *p* < 0.001, and *p* < 0.0001’, respectively.

To investigate the effect of deleting the *G22* gene on the stress tolerance of SS2, we compared the survival of SS2 strains SC19 and ∆*G22* under the stress imposed by H_2_O_2_, high-temperature, or acidic conditions. Compared with that of the SC19 and C∆*G22* strains, the survival rate of Δ*G22* was significantly lower than that of SC19 under acidic conditions, high temperatures, and in the presence of H_2_O_2_ (both *p* < 0.01; Figure 3A–C). No significant difference was found in the survival rates of three strains at a high temperature 42 °C (Figure 3C). These results indicate that *G22* is important in the bacterium’s resistance to oxidative stress and could affect the antioxidant capacity, high-temperature resistance, and acid resistance of SS2.

### 3.4. Role of G22 in Adhesion to and Invasion of Host Cells by SS2

**Figure 4:**
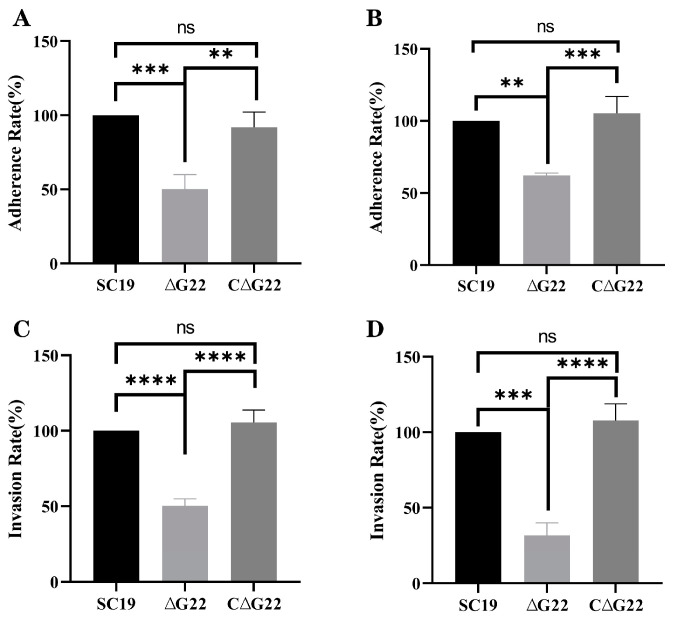
Involvement of *G22* in adhesion and invasion of host cells by SS2. (**A**) Adherence rates of SC19, ∆*G22*, and C∆*G22* in HEp-2 cells. (**B**) Adherence rates of SC19, ∆*G22*, and C∆*G22* in Caco-2 cells. (**C**) Invasion rates of SC19, ∆*G22*, and C∆*G22* in HEp-2 cells. (**D**) Invasion rates of SC19, ∆*G22*, and C∆*G22* in Caco-2 cells. Data represent the mean ± SEM of at least three independent experiments. ‘ns’, ‘**’, ‘***’, and ‘****’ indicate significant difference values with ‘*p* > 0.05, *p* < 0.01, *p* < 0.001, and *p* < 0.0001’, respectively.

The effect of *G22* deletion on the in vitro adhesion and invasion of cells by SS2 was investigated in the Caco-2 and HEp-2 cell lines. The results indicated that the adhesion of ∆*G22* to Caco-2 cells was significantly lower than that of SC19 (*p* < 0.01; Figure 4B). Similarly, the invasion of Caco-2 cells by ∆*G22* was markedly lower than that by SC19 (*p* < 0.001; Figure 4D), consistent with the results in HEp-2 cells (*p* < 0.001; Figure 4A,C). The adherence rates and invasion rates of host cells did not differ significantly between SC19 and C∆*G22*. These findings suggest that the *G22* protein contributes to the adhesion and invasion of its host cells by SS2.

### 3.5. Role of G22 in Resistance of SS2 to Host Cell Phagocytosis and Whole-Blood Resistance Assay

**Figure 5:**
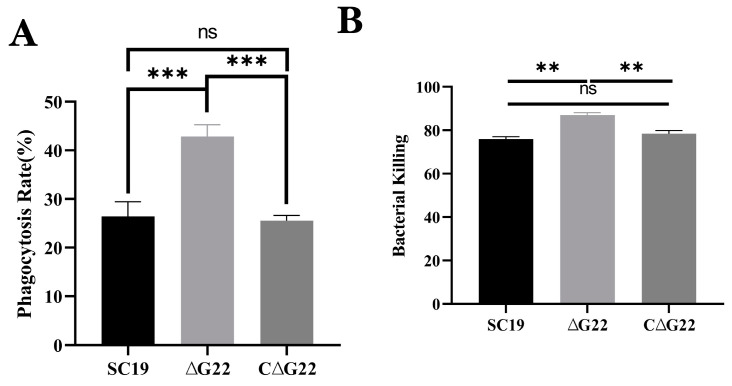
Effects of *G22* gene deficiency on pathogenicity phenotype of SS2. (**A**) Phagocytic rates of SC19, ∆*G22*, and C∆*G22* in Raw 264.7 cells. (**B**) Growth indices of SC19, ∆*G22*, and C∆*G22* in pig blood. Data represent the mean ± SEM of at least three independent experiments. ‘ns’, ‘**’, and ‘***’ indicate significantly different values with *p* > 0.05, *p* < 0.01, and *p* < 0.001, respectively.

These results showed that the phagocytosis rate of the SS2 strain ∆*G22* was significantly higher than that of the SS2 strain SC19 (*p* < 0.001). This indicates that the deletion of *G22* made the bacterium more easily phagocytosed by RAW 264.7 cells than the WT SC19 (Figure 5A) was. In a pig blood resistance assay, Δ*G22* was more vulnerable to the bactericidal effect of blood and was less viable when exposed to pig blood than strain SC19 (Figure 5B). Phagocytosis rates and bacterial killing did not differ significantly between SC19 and C∆*G22*. These results indicate that the deletion of the *G22* gene reduced the opsonic killing and pathogenic effect of SS2.

### 3.6. Knockout of G22 Significantly Reduces Mortality

**Figure 6:**
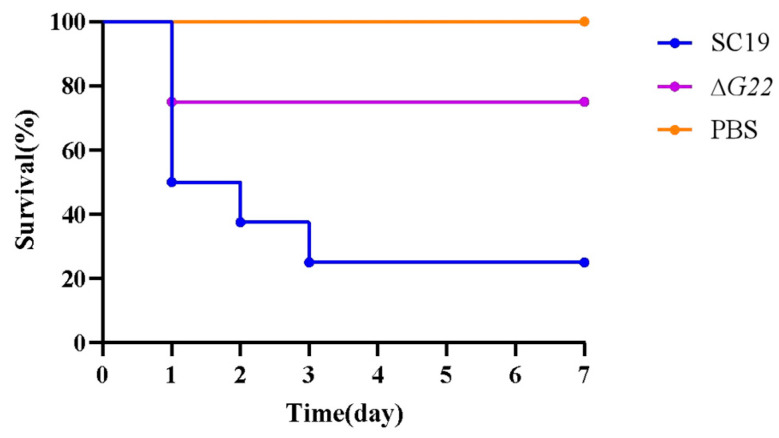
Survival curves of SC19 and ∆*G22* in a mouse infection model. Survival was analyzed using the LogRank test. Twenty-four BALB/c mice were randomly divided into three groups, with eight mice in each.

BALB/c mice were infected with 5 × 10^8^ CFU of SC19 or an equal quantity of ∆*G22*. As shown in Figure 6, 80% of mice infected with SC19 died within 3 days. In contrast, 80% of mice infected with ∆*G22* survived for 7 days postinfection. These results indicate that *G22* deficiency significantly reduced the virulence of SS2 in these mice.

### 3.7. Colonization of Mouse Tissues by WT and ∆G22 Strains

**Figure 7:**
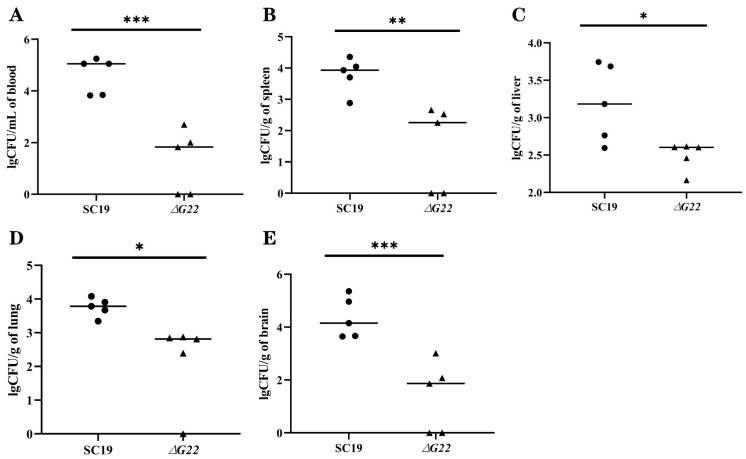
Colonization of various tissues of mice by SC19 and ∆*G22*. (**A**) Bacterial burdens in the blood of BALB/c mice at 24 h postinfection. (**B**) Bacterial burdens in spleen tissues of BALB/c mice at 24 h postinfection. (**C**) Bacterial burdens in liver tissues of BALB/c mice at 24 h postinfection. (**D**) Bacterial burdens in lung tissues of BALB/c mice at 24 h postinfection. (**E**) Bacterial burdens in brain tissues of BALB/c mice at 24 h postinfection. Data represent the mean ± SEM of at least three independent experiments. ‘*’, ‘**’, and ‘***’ indicate significantly different values with *p* < 0.05, *p* < 0.01, and *p* < 0.001, respectively.

To evaluate the inflammatory responses of animals infected with the WT and mutant strains, plasma mediators of inflammation were evaluated after 24 h of infection. The cause of the reduced virulence of ∆*G22* was investigated by determining the cytokine concentrations in the blood and the bacterial loads in different organs after infection with ∆*G22* or SC19. The numbers of SC19 cells recovered from the blood (Figure 7A) and brain (Figure 7E) were significantly higher than those of the mutant strain ∆*G22* (both *p* < 0.001). The bacterial loads of SC19 in the brain and blood were 10^5^/mL, whereas the bacterial loads of ∆*G22* in the brain and blood were < 10^3^/mL. These results indicate that resistance to the bactericidal effect of blood was significantly reduced. The spleen (Figure 7B), lung (Figure 7C), and liver (Figure 7D) samples showed similar colonization rates to the brain and blood. The bacterial loads of SC19 were higher in mice than those of ∆*G22*. These results indicate that the deletion of *G22* increased microbial clearance from the host tissues of infected mice.

### 3.8. Significant Bacterial Transcriptomic Changes with G22 Gene Deletion

**Figure 8:**
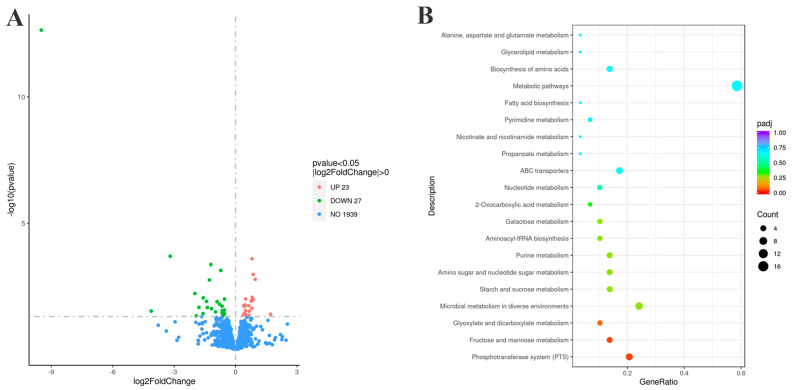
Transcriptomic profiles of strains ∆*G22* and SC19. (**A**) Volcano plot of differentially expressed genes (upregulated genes are shown in red, and downregulated genes are shown in green). (**B**) KEGG metabolic pathways of differentially expressed genes.

To investigate the molecular regulatory mechanisms of the *G22* gene in the pathogenic process of *S. suis*, we performed a comparative analysis of gene transcription levels between WT SC19 and the *G22* knockout strain (∆*G22*) using RNA sequencing technology. Our findings revealed that the deletion of *G22* resulted in the differential expression of 50 genes, with 23 genes being upregulated and 27 being downregulated (Figure 8A, Supplementary Figure S2). These differentially expressed genes (DEGs) are implicated in various biological functions, highlighting the pivotal role of *G22* in the regulation of cellular processes in *S. suis*. Gene Ontology (GO) enrichment analysis indicated that the DEGs were significantly enriched in 158 categories, predominantly related to biological processes such as sugar metabolic processes, carbohydrate transport processes, and transmembrane transport processes (Supplementary Figure S3). This suggests that *G22* plays a crucial role in modulating biological processes that influence the cellular dynamics of *S. suis*. Furthermore, Kyoto Encyclopedia of Genes and Genomes (KEGG) pathway annotation demonstrated that the DEGs were involved in 29 metabolic pathways, with a significant focus on the phosphotransferase system (PTS) and fructose and mannose metabolism, as well as glyoxylate and dicarboxylate metabolism (Figure 8B).

Specifically, the analysis of the PTS indicated that all 14 upregulated genes were associated with this system, including 4 operon genes (*manLMN*) related to mannose and the fructose-specific IIC component (*fruA*) (Figure 8B). We hypothesize that the absence of *G22* may enhance the phosphorylation of fructose and mannose, which could indirectly inhibit the phosphorylation and transport of glucose, thereby downregulating the expression of virulence factors in *S. suis* and ultimately attenuating the virulence of the SC19 strain. Furthermore, the absence of *G22* resulted in the decreased expression of *glnA* within the Two-Component System (TCS), which is implicated in the intracellular accumulation of glutamine. This alteration likely contributes to a weakened expression of associated virulence factors, leading to the attenuation of the bacterium’s virulence (Supplementary Figure S4). Additionally, the deletion of *G22* caused a reduction in the expression of *lacD* within the Quorum Sensing (QS) system, which subsequently affected the expression of the virulence factor *SpeB*, further contributing to the observed decrease in pathogenicity (Supplementary Figure S5).

In summary, transcriptomic analysis underscores the multifaceted role of the *G22* gene in regulating key biological processes and metabolic pathways, ultimately influencing the pathogenicity of *S. suis*.

## 4. Discussion

*S. suis* is a zoonotic bacterial pathogen that causes lethal infections in pigs and humans [32]. Among all the *S. suis* serotypes, SS2 is regarded as the most important and virulent zoonotic agent and is responsible for infections in swine and humans [7]. Therefore, clarifying the molecular mechanisms underlying the pathogenesis of *S. suis* is crucial in the development of novel and effective prophylactic and therapeutic strategies. Although our knowledge of the pathogenesis of *S. suis* has improved in recent years, the precise involvement of most of its putative virulence factors remains poorly understood, and further studies are required to clarify it [33].

In this study, we defined the phenotypic differences between strains SC19 and ∆*G22* to characterize the function of *G22* in terms of the pathogenicity of SS2, to extend our understanding of this zoonotic pathogen. Comparative growth curves showed that the deletion of the *G22* gene had no effect on bacterial growth. However, it reduced the cytotoxicity, adhesion, and invasion of SC19 in Caco-2 and HEp-2 cells, indicating that *G22* contributes to the adhesion to and invasion of host cells by SS2. *G22* deletion also rendered the bacterium more easily phagocytosed by RAW 264.7 cells than SC19. In a pig blood resistance assay, Δ*G22* was more vulnerable to the bactericidal effect of blood than SC19 and was less viable when exposed to pig blood. The deletion of *G22* reduced the antioxidant capacity and acid resistance of SS2. The *G22* protein mediates SS2 infection by affecting bacterial invasion, phagocytosis resistance, and virulence. This evidence suggests that *G22* is one of the multifunctional factors that play an essential role in the virulence and pathogenesis of SS2. A previous study showed that the *lacD* gene is generally distributed in SS2 strains, which encodes a novel metabolism-related factor that contributes to stress tolerance, antimicrobial resistance, and virulence modulation, as moonlighting roles in SS2 [21]. As the most important virulence factor of *S. suis*, Cps also prevents host phagocytes from phagocytizing and killing the bacterium [34]. There are several similarities between *G22* and the abovementioned virulence factors. However, the pathogenesis of the *G22* virulence factor requires further investigation in future research.

As previously reported, infected mice developed the typical clinical symptoms of *S. suis* disease, including septicemia, meningitis, and septic shock, followed by clinical signs of central nervous system (CNS) dysfunction [35]. We experimentally infected mice to evaluate the inflammatory response of animals infected with strains SC19 and ∆*G22*. The results indicated that the normal resistance to the bactericidal effect of blood and the BBB-damaging ability of SC19 were significantly reduced in ∆*G22* and that the deletion of *G22* increased microbial clearance from the tissues of infected mice. The survival rate of strain Δ*G22* in a microenvironment of H_2_O_2_-induced oxidative stress was also greatly reduced compared with that of SC19. Consistent with previous findings, the reduced tolerance of ∆*G22* for oxidative stress may be an important factor in the reduced survival of the mutant in infected mouse tissues, because ∆*G22* is probably less well adapted to the host environment during infection [26]. This is consistent with our experimental finding that the BBB-damaging ability of ∆*G22* was significantly reduced.

The SS2 biofilm structure is complex and may contain lipoprotein, Cps, and host-derived fibrin, although most bacterial biofilms are predominantly composed of a polysaccharide matrix. Extracellular polysaccharides can be categorized into different types based on their apparent morphology: mucin-like protein (also known as ‘mucoid’), which generally creates a resilient outer membrane that adheres to the cell surface through intermolecular hydrogen bonds and other non-covalent bonds, also known as ‘Cps’ [36]. The Cps of SS2 strains is composed of glucose, galactose, N-acetylglucosamine, rhamnose, and sialic acid, which are synthesized by the capsule synthesis clusters [37]. Transmission electron microscopic analysis of SC19 and ∆*G22* showed that the capsular structure of ∆*G22* had irregular matrix deposition with visible spatial discontinuities. This differs from previous reports, in which the capsule of the deletion mutant strain was thinner than that of the WT [37,38].

We investigated the transcriptome-level changes in SC19 in response to the deletion of *G22* to determine how *G22* regulates the high-level pathogenicity of SS2. The deletion of *G22* led to the differential expression of 50 genes, among which several encoded potential virulence-associated factors. The first was associated with the phosphotransferase system (PTS). The *G22* deletion altered the expression of fourteen PTS-related genes, all of which were upregulated. These included four genes in the mannose-specific PTS (*manL*, *manM*, *manN*, and *manO*), as well as the fructose-specific IIC component gene *fruA*. Therefore, we speculate that the deletion of *G22* promoted the phosphorylation of fructose and mannose [39,40,41], thereby indirectly inhibiting the phosphorylation and transport of glucose, inhibiting the expression of virulence factors, and ultimately reducing the virulence of *S. suis* SC19. The deletion of *G22* also reduced the expression of *glnA* in the two-component system (TCS), which, we speculate, affected the accumulation of intracellular glutamine in *S. suis*, weakening the expression of related virulence factors and ultimately reducing the virulence of *S. suis* [42,43]. The third virulence-associated system affected was the quorum sensing system (QSS). The loss of *G22* reduced the expression of *lacD* in the QSS, which affected the expression of the virulence factor *SpeB*, also weakening bacterial virulence. Therefore, we investigated whether *G22* is involved in the virulence of SS2 [21,44] because there is sparse experimental evidence of the biological function of *G22* in SS2 pathogenicity. Our integrative omics analyses reveal that secreted protein *G22* is a master virulence coordinator in *S. suis*. Key findings demonstrate that *G22* deletion triggers capsular disorganization (30% reduction in CPS thickness), impaired host interaction, and compromised stress resistance, concurrent with the significant upregulation of PTS components and glycolytic genes. This tripartite regulatory pattern suggests that *G22* functions as a metabolic–capsular coupler—a novel mechanism where extracellular capsule biogenesis is energetically coupled with intracellular carbon flux via PTS-mediated ATP generation. While analogous nutrient sensing systems exist in Klebsiella pneumoniae [45], the direct linkage of CPS synthesis to sugar phosphotransferase activity represents an evolutionary innovation in *S. suis* pathogenicity. Future studies should delineate whether *G22* acts as a PTS substrate chaperone or phosphatase modulator to bridge these pathways.

In addition to the findings presented in this study, several limitations should be acknowledged. Firstly, although mouse models and cell cultures were used to investigate the pathogenicity and virulence of SS2, there may be differences between these models and natural infections in pigs or humans. Therefore, the results obtained from animal experiments and cell cultures may not fully reflect the actual situation in vivo. Secondly, the study focused on a specific virulent strain, SC19, which may limit the generalizability of the findings to other strains or serotypes of *S. suis*. Future studies should explore the role of the *G22* gene in different strains and serotypes to validate the current findings. Thirdly, the pathogenesis of *S. suis* infections is complex and involves multiple factors. The current study only explored the role of the *G22* gene, and other genes and proteins may also play crucial roles in the virulence and pathogenesis of *S. suis*. Therefore, further research is needed to elucidate the entire pathogenic mechanism.

## Figures and Tables

**Table 1:** Strains and plasmids used in this study.

Strain or Plasmid	Characteristics and Functions	Sources or Reference
SC19	Virulent strain isolated from the brain of a dead pig; Serotype 2	Laboratory collection
∆*G22*	∆*G22*-deletion mutant strain	This study
C∆*G22*	Complemented strain of *G22*, Spc^R^, Cm^R^	This study
*Escherichia coli* DH5α	Cloning host for recombinant vector	Purchased from Shanghai Sangon Biotech Co., Ltd., Shanghai, China
pSET4s	Temperature-sensitive *E. coli*-*S. suis* shuttle vector, Spc^R^	Purchased from Shanghai Ke Lei Biological Technology Co., Ltd., Shanghai, China
pSET2	*E. coli*-Streptococcus shuttle cloning vectors; Spc^R^	Laboratory collection
pSET4s::*G22*	A recombinant vector with the background of pSET4S, designed for Δ*G22*, Spc^R^	This study
pSET2::*G22*	pSET2 containing the *G22* gene and its promoter, Spc^R^	This study

Spc^R^, Spectinomycin resistant; Cm^R^, Chloramphenicol resistance.

**Table 2:** Oligonucleotide primers used in this study.

Primer	Primer Sequence (5′-3′)	Function or PCR Product
*G22*L-F	TTGTAAAACGACGGCCAGTGAATTCGAAGCAATCTGTCGTGGAGTTG	Upstream fragment amplification of *G22*
*G22*L-R	ATTACTATCCACGTTTCATTTTTGAAATATCTCC	
*G22*R-F	AATGAAACGTGGATAGTAATTCAGTTTTG	Downstream fragment amplification of *G22*
*G22*R-R	CTATGACCATGATTACGCCAAGCTTCCACTGTTCTCTATCCATATG	
W1	TTGTAAAACGACGGCCAGTGAATTCGAAGCAATCTGTCGTGGAGTTG	Identification outside the homologous region
W2	CTATGACCATGATTACGCCAAGCTTCCACTGTTCTCTATCCATATG	
N1	AAGTTGGTCTGTGTGCTATGG	Internal fragment of *G22* identification
N2	TTAGAACCAGCAGCTCTCG	

Note: Underlining indicates homology with the pSET4s vector.

## Data Availability

The raw sequencing datasets from this study have been curated and deposited in the Sequence Read Archive of the National Center for Biotechnology Information (NCBI) under accession number PRJNA1130436, promoting data sharing and reproducibility in future research. The original contributions presented in the study are included in the article, further inquiries can be directed to the corresponding authors.

## Supplementary Materials

The following supporting information can be downloaded at: https://www.mdpi.com/article/10.3390/microorganisms13040774/s1, Supplementary Figure S1. Comparison of sequences of SC19 and ∆*G22*. The consensus sequences are indicated in red. Supplementary Figure S2. Hierarchical cluster diagram of differential genes of SC19 and ∆*G22*.The expression genes are clustered in rows in each heatmap, and the isolates are shown in columns. The numbers in the end in isolates representing each of the three biological replica. High expression genes are represented in red and low expression genes are represented in blue. Supplementary Figure S3. The significantly enriched GO terms of the DEGs of SC19 and ∆*G22*. The top 30 significantly enriched GO terms are shown. The X-axis indicates the enriched GO terms, and the Y-axis indicates the number of the DEGs for each GO term. The GO terms in red color belonged to biological processes, those in green belonged to cellular components, and those in blue belonged to molecular function. The GO terms with a corrected *p*-value < 0.05 were considered to be significantly enriched. Supplementary Figure S4. KEGG metabolic pathway for the Two-Component System (TCS). Low expression genes are highlighted in green. Supplementary Figure S5. KEGG metabolic pathway for the Quorum Sensing (QS) system. Low expression genes are highlighted in green.
