# Supplementary material for: Mechanisms Underlying the Effects of Secretory Protein G22 on Biological Characteristics and Virulence of Streptococcus suis
Source: Microorganisms. 2025 Mar 28;13(4):774. doi: 10.3390/microorganisms13040774 (PMC12029192; doi:10.3390/microorganisms13040774)
Supplement: Supplementary file 1 [file microorganisms-13-00774-s001.zip › microorganisms-3482715-supplementary.pdf]

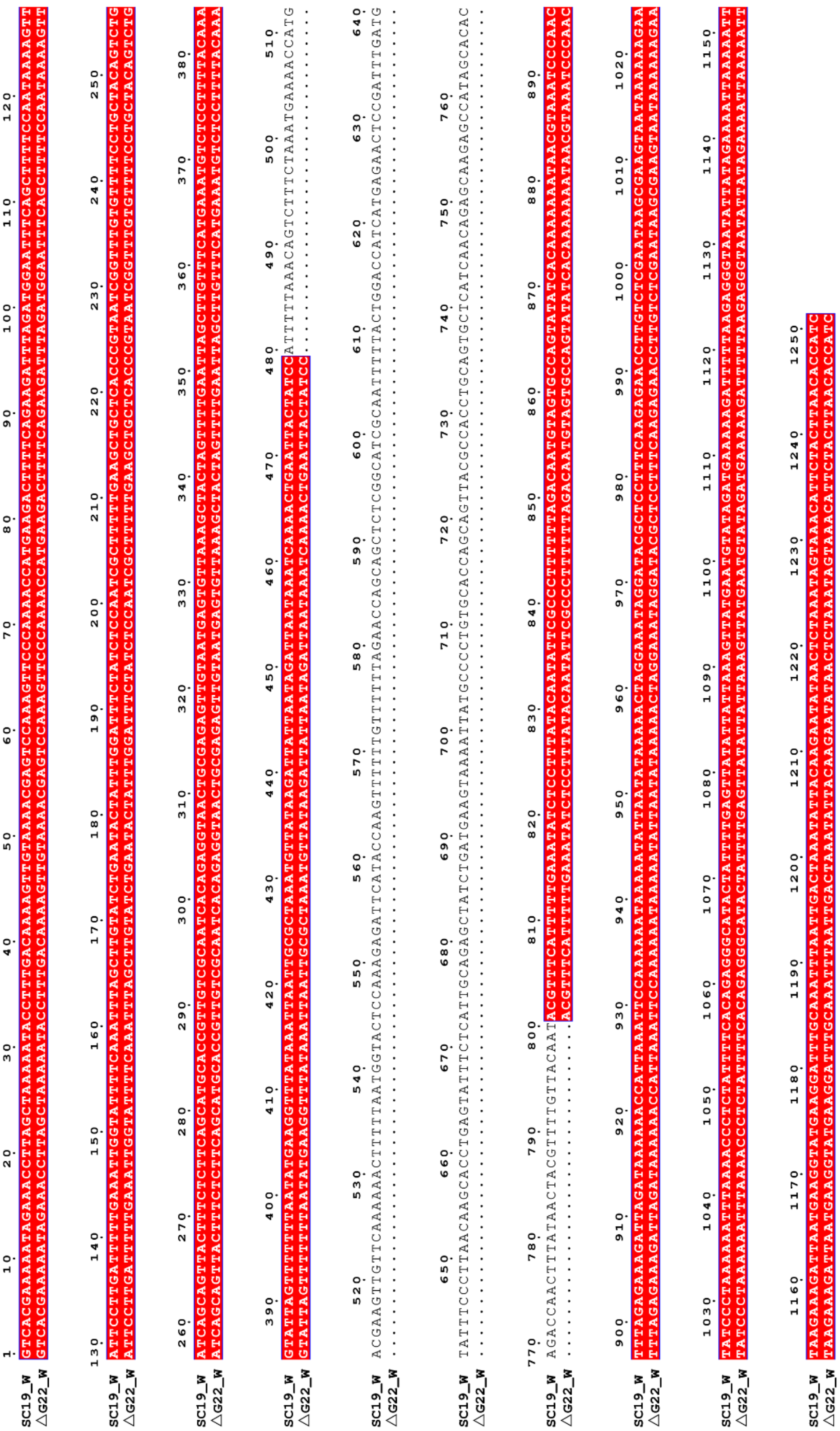

**Supplementary Figure S1.** Comparison of sequences of SC19 and ΔG22. The consensus sequences are indicated in red.

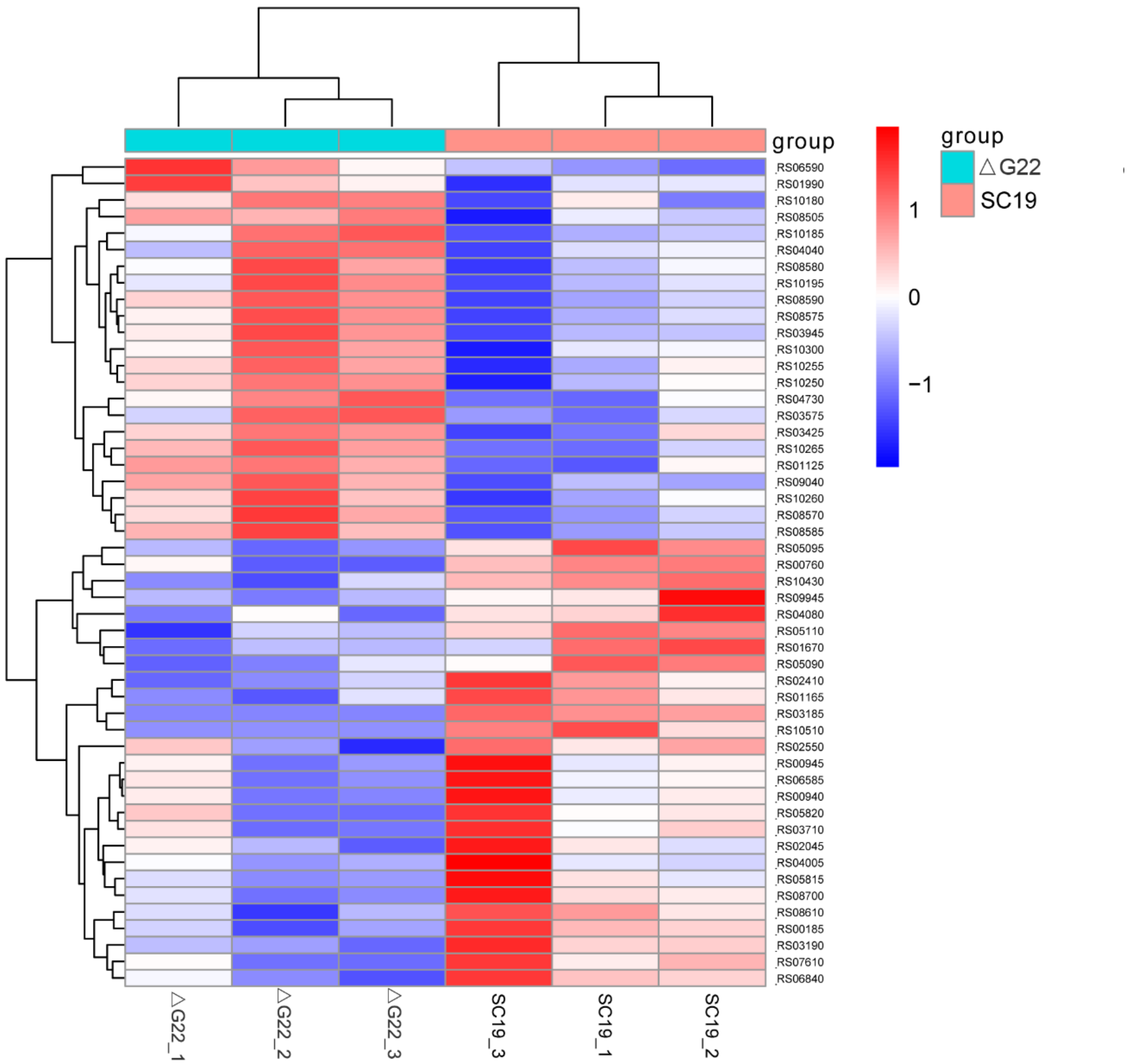


**Supplementary Figure S2.** Hierarchical cluster diagram of differential genes of SC19 and ΔG22.The expression genes are clustered in rows in each heatmap, and the isolates are shown in columns. The numbers in the end in isolates representing each of the three biological replica. High expression genes are represented in red and low expression genes are represented in blue.

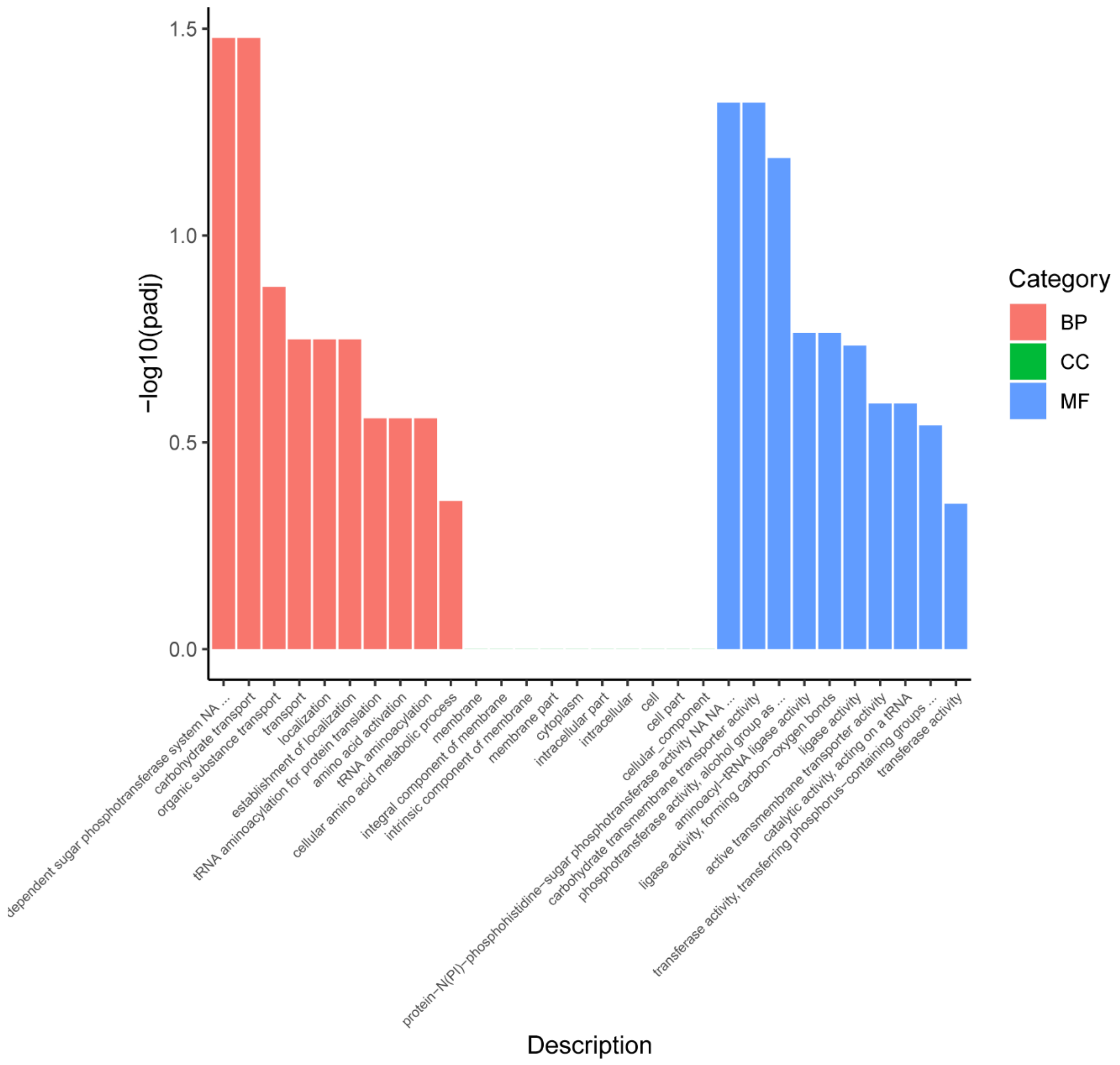


**Supplementary Figure S3.** The significantly enriched GO terms of the DEGs of SC19 and ΔG22. The top 30 significantly enriched GO terms are shown. The X-axis indicates the enriched GO terms, and the Y-axis indicates the number of the DEGs for each GO term. The GO terms in red color belonged to biological processes, those in green belonged to cellular components, and those in blue belonged to molecular function. The GO terms with a corrected p-value < 0.05 were considered to be significantly enriched.

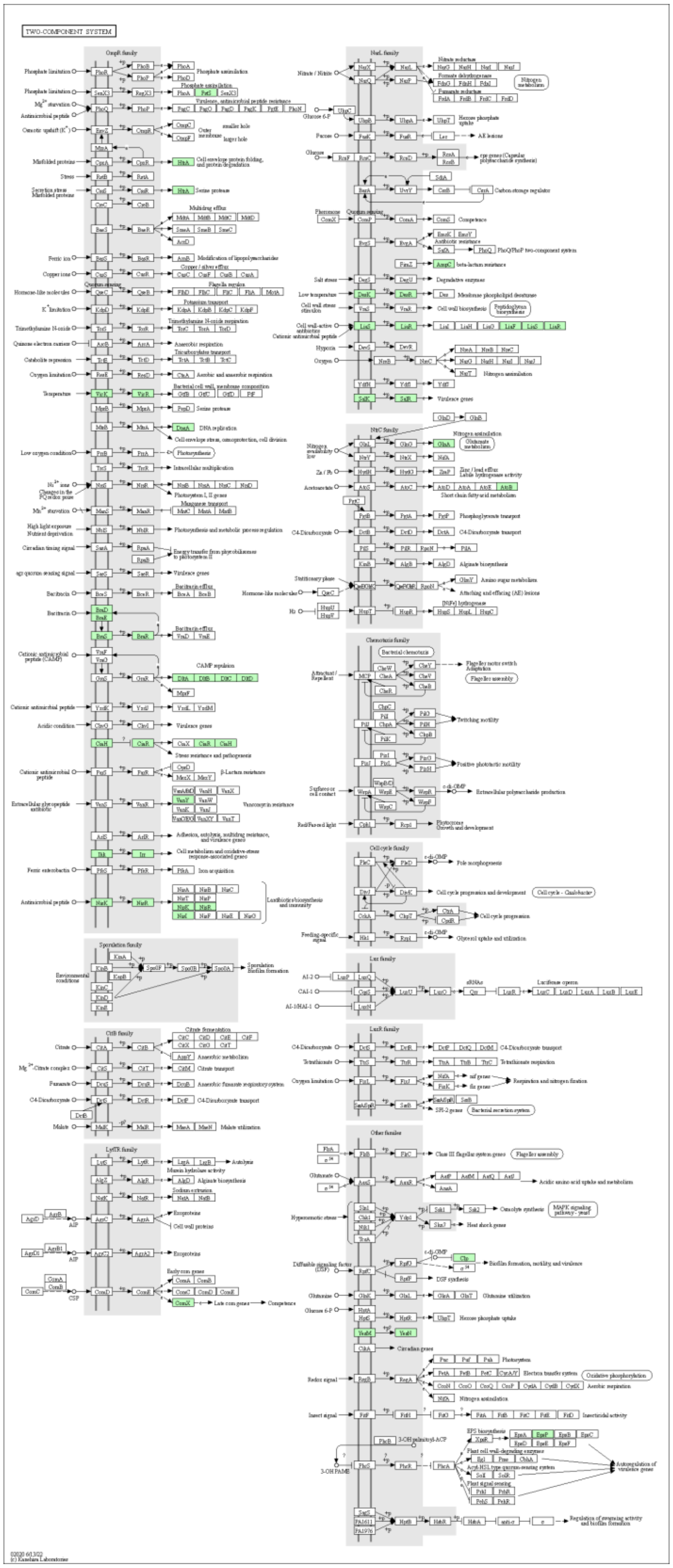


**Supplementary Figure S4.** KEGG metabolic pathway for the Two-Component System (TCS). Low expression genes are highlighted in green.

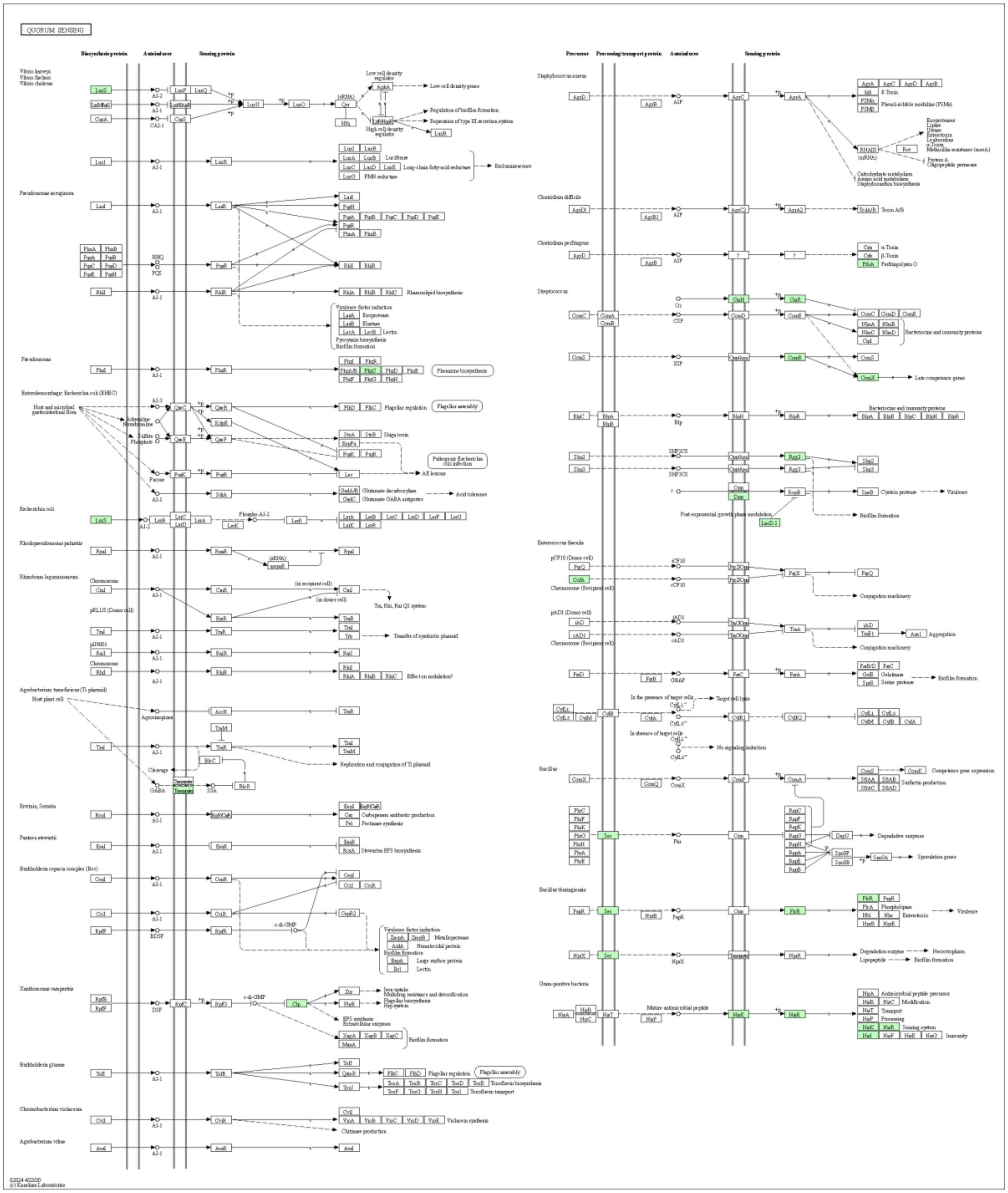


**Supplementary Figure S5.** KEGG metabolic pathway for the Quorum Sensing (QS) system. Low expression genes are highlighted in green.
